# Genome-wide characterization of the *Bursaphelenchus xylophilus UGT* gene family and its potential roles in detoxification and host interaction

**DOI:** 10.3389/fpls.2026.1786843

**Published:** 2026-04-10

**Authors:** Xiong Xiong, Jie Li, Shuaibin Sun, Kunyang Ju, Chengming Yu, Yehan Tian, Jinhua Xu, Gengwu Sun, Chuanrong Li, Huixiang Liu

**Affiliations:** 1 College of Forestry, Shandong Agricultural University, Tai’an, China; 2 College of Plant Protection, Shandong Agricultural University, Tai’an, China; 3 Weihai Wendeng District Forestry Development Center, Weihai, China

**Keywords:** *Bursaphelenchus xylophilus*, detoxification, expression profile, phylogenetic analysis, UGT gene family

## Abstract

**Introduction:**

Pine wilt disease caused by *Bursaphelenchus xylophilus* constitutes a severe threat to global pine forests. The UDP-glycosyltransferase (*UGT*) gene family is involved in xenobiotic detoxification, environmental adaptation, and other biological processes, but systematic identification of this family in *B. xylophilus* has not yet been performed. This study aimed to comprehensively identify *UGT* family members in *B. xylophilus* and clarify their molecular roles in xenobiotic detoxification and host adaptation regulation.

**Methods:**

Based on genome-wide data, we identified 47 *UGT* genes in *B. xylophilus* and systematically analyzed their phylogenetic relationships, physicochemical properties, gene structures, transcription factor binding sites, as well as expression profiles under nematicide stress and during the infection stage.

**Results:**

The results showed that the *UGT* genes are distributed across 5 chromosomes. Compared with those of *Caenorhabditis elegans*, these genes exhibit both conserved and species-specific evolutionary patterns, and no gene duplication events were detected in the *UGT* genes. Expression profile analysis revealed that members of this gene family are involved in the stress response of *B. xylophilus* to nematicide exposure; most *UGT* genes were significantly upregulated during the infection stage, and several genes maintained high expression levels, suggesting that they may play key roles in xenobiotic detoxification and host adaptation.

**Discussion:**

This study clarifies the functional importance of the *UGT* gene family in *B. xylophilus*, providing a theoretical basis for elucidating its adaptive mechanisms and developing control strategies.

## 1 Introduction

Pine wilt disease (PWD) is one of the greatest threats to pine forests worldwide, causing economic losses and disrupting forest ecosystems in Japan and China (Yu et al., 2025). *Bursaphelenchus xylophilus* (Steiner & Buhrer) Nickle, the pathogen of PWD, exhibits remarkable adaptability, with rapid colonization and high reproductive capacity that facilitate disease spread and makes effective control challenging (Zhou et al., 2024). Despite extensive ecological and physiological studies, the molecular mechanisms underlying its environmental tolerance, chemical detoxification, and host adaptation remain largely unexplored, understanding these mechanisms is crucial for developing targeted strategies to mitigate PWD and manage the impact of this invasive species.

UDP-glycosyltransferases (UGTs) are a large and evolutionarily conserved superfamily of enzymes that catalyze the transfer of glycosyl groups from UDP-activated sugar donors to a variety of acceptor molecules, including hormones, secondary metabolites, and xenobiotics (Meech et al., 2019). This glycosylation process typically increases the solubility and stability of hydrophobic compounds, thereby promoting their detoxification, transport, and excretion. In invertebrates, UGTs have been shown to participate in xenobiotic detoxification, cuticle biosynthesis, hormone regulation, and adaptation to environmental stress (Ahn et al., 2021). In *B. xylophilus*, *UGT* genes are believed to play vital roles in the nematode’s adaptation to its complex chemical environment within pine hosts. Transcriptomic analyses have demonstrated that several *UGT* genes are significantly upregulated under host-derived terpenoid stresses, such as β-pinene, suggesting their involvement in metabolizing host defense compounds (Li et al., 2020). Furthermore, genome-wide studies revealed a notable expansion of *UGT* and other detoxification-related gene families, which may underpin the nematode’s remarkable ecological adaptability and invasiveness (Zhang et al., 2020). These findings underscore the importance of *UGT* genes in the detoxification network of *B. xylophilus* and their potential contribution to host adaptation.

Despite these advances, the comprehensive characterization of the *UGT* gene family in *B. xylophilus* remains limited. Previous studies have mostly focused on transcriptomic responses under specific stresses, without systematically analyzing gene structure, evolutionary relationships, conserved motifs, and potential regulatory mechanisms. Furthermore, how *UGT* genes respond across different physiological conditions. In this study, we performed a genome-wide identification and characterization of *UGT* genes in *B. xylophilus*. A total of 47 *UGT* genes were identified and analyzed in terms of their phylogenetic relationships, conserved domains, gene structures, and regulatory elements. We further investigated their expression profiles under nematicide stress and infection stages based on transcriptomic datasets. This work provides a novel insight into the molecular basis of xenobiotic detoxification and host adaptation in *B. xylophilus*, contributing to a deeper understanding of its survival strategies and offering potential molecular targets for managing PWD.

## 2 Materials and methods

### 2.1 Identification of *UGT* genes in *B. xylophilus*

*UGT* genes were identified using the TS-1 genome assembly (Li et al., 2023). The hidden Markov model (HMM) profile of the UGT domain (Pfam: PF00201) was obtained from the Pfam database (http://pfam.xfam.org/). Genome-wide searches were conducted with HMMER v3.3.2 (E-value ≤ 1×10^-5^) to identify putative UGT candidates (Finn et al., 2011). Candidates were validated for the presence of the conserved UGT domain using the NCBI Conserved Domain Database (https://www.ncbi.nlm.nih.gov/) and SMART (https://smart.embl.de/). After removing redundant sequences and incomplete domains, the validated *UGT* genes were subjected to downstream analyses. Physicochemical properties of UGT proteins including amino acid length, molecular weight, isoelectric point, and GRAVY values were calculated using ExPASy ProtParam (https://web.expasy.org/protparam/). WoLF PSORT (https://wolfpsort.hgc.jp) was used to predicted subcellular localization, the 3D structures of UGT proteins were visualized using AlphaFold3 (Abramson et al., 2024).

### 2.2 Phylogenetic analysis of *UTG* genes

To investigate evolutionary relationships, UGT protein sequences from *Caenorhabditis elegans* were retrieved from WormBase (https://wormbase.org/) and UniProt (https://www.uniprot.org/). Redundant and unusually short sequences were excluded. Multiple sequence alignment was performed using MAFFT v7.505 with default parameters (Katoh et al., 2002). A phylogenetic tree was constructed in IQ-TREE using the Maximum Likelihood (ML) method with 2000 bootstrap replicates to assess branch support and was visualized using iTOL (Nguyen et al., 2015; Letunic and Bork, 2024).

### 2.3 Phylogenetic and structural analysis of *B. xylophilus UGT* genes

Phylogenetic tree of *B. xylophilus UGT* genes was reconstructed following the 2.1 procedure. Conserved motifs were predicted using MEME (https://meme-suite.org/meme/tools/meme) (Bailey et al., 2015), structural domains were annotated via CDD and UniProt, and functional domain information was extracted from the TS-1 genome.

### 2.4 Chromosomal localization

The chromosomal locations of *UGT* genes were determined based on the gene location visualization function of the GTF/GFF files from the TS-1 genome. Gene coordinates were mapped to the chromosomes using TBtools-II (Chen et al., 2020), and the resulting distribution was visualized to assess genomic organization. Gene density along the chromosomes was calculated and illustrated to reveal clustering patterns. Gene duplication events and collinear relationships among *UGT* genes were analyzed and visualized using TBtools-II.

### 2.5 Transcription factor binding site prediction

The 2 kb upstream sequences of all *UGT* genes were extracted, relative to their translation start sites. Potential transcription factor (TF) binding sites were predicted based on the *C. elegans* TF database from JASPAR (https://jaspar.elixir.no/). Motif discovery and scanning were performed using MEME and FIMO (https://meme-suite.org/meme/tools/fimo), respectively, with a p-value threshold of 1×10^-5^. Predicted TF binding sites were compiled and visualized in TBtools-II to illustrate the distribution of potential regulatory elements.

### 2.6 Transcriptome data sources and analysis

For the host infection transcriptome, approximately 10,000 nematodes were inoculated onto three-year-old *Pinus thunbergii* seedlings. After 15 days of infection, nematodes were isolated from infected pine tissues. Each treatment included three biological replicates. Nematodes cultured on *Botrytis cinerea* under laboratory conditions were used as the control group. RNA extraction and sequencing were performed by OE Biotech Co., Ltd. (Shanghai, China) using the Illumina NovaSeq platform. For pesticide treatments, the transcriptome data under emamectin benzoate stress were derived from NCBI database (BioProject: PRJNA1401456), while the transcriptome data under tetramycin B3 stress were obtained from previously published studies (Sun et al., 2024). Low-quality bases and adapter sequences were removed using Trimmomatic (Bolger et al., 2014). Clean reads were aligned to the *B. xylophilus* TS-1 reference genome using HISAT2 (Kim et al., 2019), and transcript abundance was quantified as fragments per kilobase of transcript per million mapped reads (FPKM) using StringTie (Pertea et al., 2015). Differentially expressed genes (DEGs) were identified using DESeq2 with the thresholds |log_2_ (fold change) ≥ 1.5 and adjusted p-value < 0.05 (Love et al., 2014). Heatmaps illustrating the expression profiles of *UGT* genes were generated using R v4.5.2, based on z-score–normalized FPKM values.

### 2.7 Real-time quantitative polymerase chain reaction detection

Total RNA was extracted using a Trizol reagent (Invitrogen). RNA quality was verified by agarose gel electrophoresis and spectrophotometric analysis (A260/A280 ratio). RNA (2 μg) was converted into cDNA using the HiScript Il 1st Strand cDNA Synthesis Kit (+gDNA wiper) (Vazyme). RT-qPCR was accomplished using the PerfectStart Green qPCR SuperMix (TransGen Biotech) in the LightCycler 96 real-time PCR system (Roche). *actin-F* and *actin-R* were used as endogenous controls (Wang et al., 2013). We used melting curves to monitor non-specific amplifications. Relative expression level was computed using 2^-ΔΔCt^ method. The primer sequences used are listed in supplemental **Table 1**.

**Table 1:** Biophysical properties and subcellular localization of the *UGT* genes in *B. xylophilus*.

Gene	ID	AA number	MW	pI	II	AI	GRAVY	Subcell Loc
*BXugt01*	*BX01G1734*	538	62208.64	9.2	33	93.55	-0.146	Plasma membrane
*BXugt02*	*BX02G1613*	510	58283.21	9.42	36.86	88.73	-0.167	Vacuole
*BXugt03*	*BX02G1661*	525	59595.28	6.71	37.54	96.8	0.052	Chloroplast
*BXugt04*	*BX02G2078*	520	58896.52	8.09	37.2	91.48	-0.023	Plasma membrane
*BXugt05*	*BX02G2164*	519	58711.42	8.5	35.56	91.52	-0.033	Cytoplasm
*BXugt06*	*BX02G2195*	693	78513.34	5.54	41.7	89.64	-0.13	Cytoplasm
*BXugt07*	*BX02G2210*	487	55081.87	6.64	31.84	102.03	0.072	Cytoplasm
*BXugt08*	*BX02G2212*	526	59962.31	8.4	31.88	97.45	-0.044	Vacuole
*BXugt09*	*BX02G2213*	530	60196.92	8.57	24.73	93.96	-0.017	Cytoplasm
*BXugt10*	*BX02G2436*	1125	128352.66	7.82	48.56	73.87	-0.571	Nucleus
*BXugt11*	*BX03G0581*	321	36310.04	5.86	39.99	102.93	0.06	Chloroplast
*BXugt12*	*BX03G0582*	200	22574.29	9.22	27.84	111.6	0.028	Golgi apparatus
*BXugt13*	*BX03G0584*	516	58446.5	6.35	31.58	95.41	-0.052	Chloroplast
*BXugt14*	*BX03G0585*	498	56723.63	6.16	34.07	101.79	-0.036	Plasma membrane
*BXugt15*	*BX03G0647*	528	60114.71	8.35	35.82	92.61	-0.029	Chloroplast
*BXugt16*	*BX03G0648*	526	60306.87	6.55	31.23	93.57	-0.117	Chloroplast
*BXugt17*	*BX03G0677*	174	19945.98	5.34	36.71	91.9	0.111	Cytoplasm
*BXugt18*	*BX03G0678*	1508	171409.2	6.31	40.63	84.05	-0.376	Plasma membrane
*BXugt19*	*BX03G0764*	553	62882.35	6.15	42.2	98.55	-0.091	Chloroplast
*BXugt20*	*BX04G1048*	508	57911.38	9.2	30.59	95	0.008	Chloroplast
*BXugt21*	*BX05G0252*	521	58932.08	9	32.01	89.85	-0.024	Plasma membrane
*BXugt22*	*BX05G0437*	525	59437.17	8.9	37.02	97.89	0.03	Plasma membrane
*BXugt23*	*BX05G0561*	644	74257.74	9.17	32.16	86.91	-0.178	Vacuole
*BXugt24*	*BX06G0625*	544	62215.93	8.91	40.13	97.81	-0.153	Plasma membrane
*BXugt25*	*BX06G1038*	2754	318406.06	6.2	39.44	85.25	-0.486	Extracellular
*BXugt26*	*BX06G1737*	516	59393.97	7.22	32.94	80.33	-0.181	Chloroplast
*BXugt27*	*BX06G1796*	783	88752.56	9.19	33.94	103.08	0.168	Plasma membrane
*BXugt28*	*BX06G1846*	551	62394.16	9.4	32.77	106.68	0.018	Plasma membrane
*BXugt29*	*BX06G1890*	512	58615.79	9	37.81	88.71	-0.096	Vacuole
*BXugt30*	*BX06G1904*	1138	127677.99	8.51	38.21	96.93	-0.015	Plasma membrane
*BXugt31*	*BX06G1974*	530	59287	8.75	37.94	99.53	0.045	Nucleus
*BXugt32*	*BX06G1975*	503	56505.3	8.45	34.03	90.12	-0.126	Cytoplasm
*BXugt33*	*BX06G2108*	521	58730.61	9.03	32.4	97.72	-0.003	Plasma membrane
*BXugt34*	*BX06G2110*	383	43876.55	8.16	34.03	92.11	-0.111	Cytoplasm
*BXugt35*	*BX06G2111*	1031	116026.42	8.51	34.72	94.5	-0.019	Plasma membrane
*BXugt36*	*BX06G2112*	532	60019.19	6.21	38.64	92.05	-0.043	Plasma membrane
*BXugt37*	*BX06G2113*	521	58792.08	6.29	33.51	92.51	-0.037	Chloroplast
*BXugt38*	*BX06G2114*	556	62424.49	6.24	32.72	101.69	0.078	Plasma membrane
*BXugt39*	*BX06G2132*	543	61493.36	8.89	33.55	93.7	-0.067	Nucleus
*BXugt40*	*BX06G2136*	529	60217.27	8.85	37.25	97.66	-0.016	Plasma membrane
*BXugt41*	*BX06G2223*	1528	172945.87	8.79	37.15	95.12	-0.097	Plasma membrane
*BXugt42*	*BX06G2270*	531	60457.94	8.66	28.44	92.49	-0.124	Cytoplasm
*BXugt43*	*BX06G2273*	520	59311.6	7.72	30.41	95.19	-0.022	Vacuole
*BXugt44*	*BX06G2301*	527	59517.59	5.92	34.54	94.16	-0.057	Plasma membrane
*BXugt45*	*BX06G2362*	533	60464.61	9.39	29.95	94.88	0.028	Vacuole
*BXugt46*	*BX06G2363*	535	59885.56	8.86	35.59	92.22	0.094	Chloroplast
*BXugt47*	*BX06G2365*	536	59720.52	8.9	35.73	95.88	0.127	Chloroplast

AA number, amino acid number; MW, molecular weight; pI, isoelectric point; II, instability index; AI, aliphatic index; GRAVY, grand average of hydropathicity; Subcell Loc, subcellular localization.

## 3 Results

### 3.1 Identification, physicochemical characterization and chromosomal localization of *UGT* genes in *B. xylophilus*

**Figure 1:**
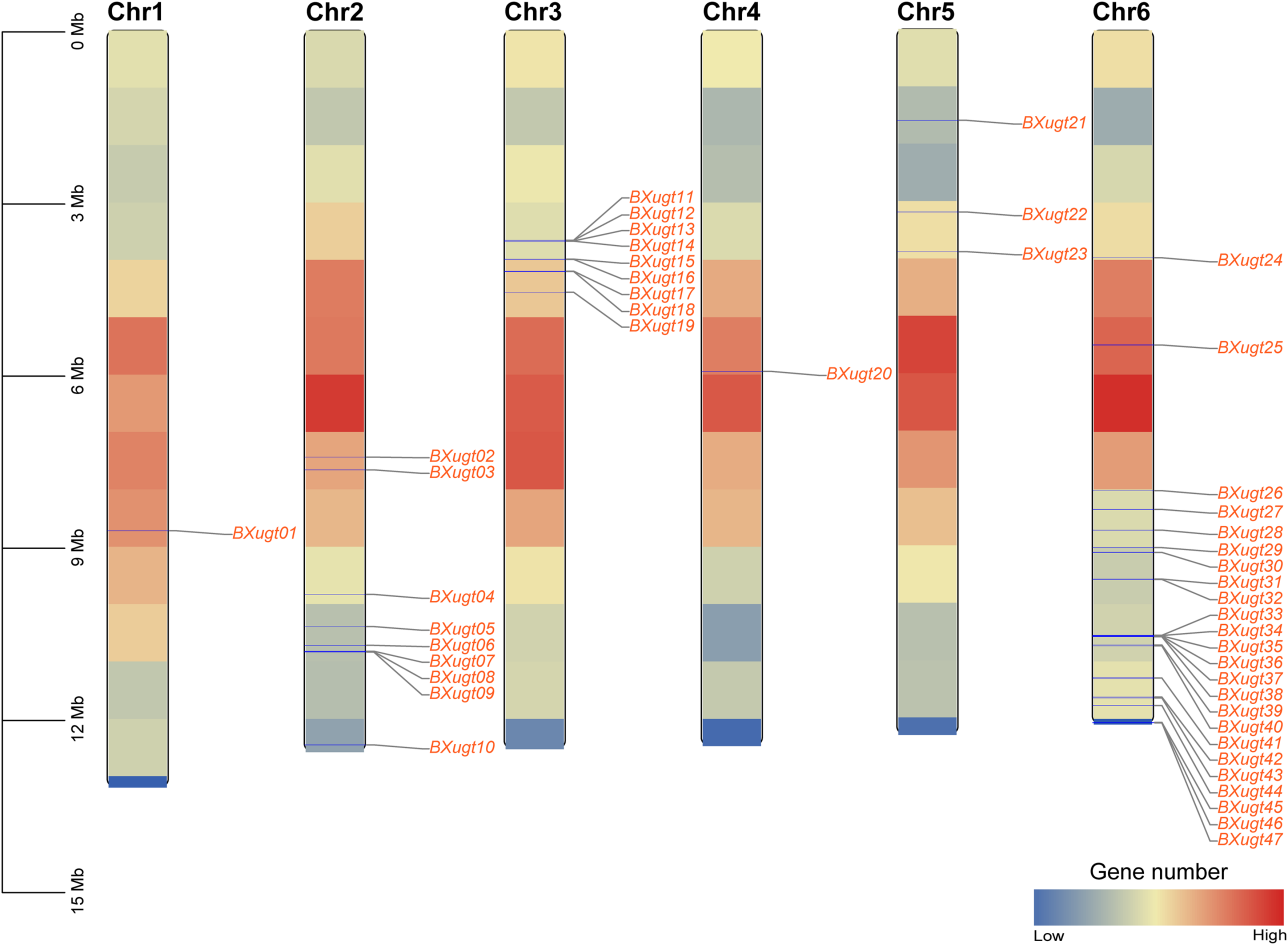
Chromosomal localization of *UGT* genes in *B. xylophilus*. Chromosome colors indicate gene density, with red representing higher gene density and blue representing lower gene density.

A total of 47 *UGT* genes were identified in the genome of *B. xylophilus*. For the convenience of subsequent analysis, *UGT* genes in *B. xylophilus* were renamed *BXugt01*–*BXugt47* according to their arrangement order on the chromosomes. The deduced UGT proteins exhibit marked variation in amino acid length, molecular weight (MW), isoelectric point (pI), instability index (II), aliphatic index (AI), grand average of hydropathicity (GRAVY), and predicted subcellular localization (Subcell Loc) (**Table 1**). The number of UGT*s* amino acids (AA number) from 174 to 2754, with corresponding molecular weights from 19.95 kDa to 318.41 kDa and theoretical pI values between 5.34 and 9.42, calculated GRAVY values varied from –0.571 to +0.168, suggesting that most UGT proteins are hydrophilic, while a few may be membrane-associated. Subcellular localization indicated that the majority of UGT proteins were localized to the plasma membrane, chloroplast, and cytoplasm, followed by fewer proteins in the vacuole and nucleus. A limited number of proteins were predicted to reside in the endoplasmic reticulum, Golgi apparatus, mitochondria, or extracellular space. Chromosomal localization analysis revealed that the *UGT* genes are unevenly distributed across 6 chromosomes. Most *UGT* genes are located in regions with relatively low gene density. Among them, chromosome 5 contained the largest number of *UGT* genes, although it was not the longest chromosome. In contrast, relatively few *UGT* genes were located on chromosomes 1, 4, and 5. Clusters of *UGT* genes were observed on chromosomes 2, 3, and 6 (**Figure 1**). To further elucidate the evolutionary mechanisms of the *UGT* gene family, this study analyzed the gene expansion patterns of this family in the *B. xylophilus* genome, with a specific focus on investigating tandem arrangement and segmental duplication events. The results showed that a total of 8 pairs of tandemly arranged *UGT* genes were identified in the *B. xylophilus* genome, namely *BXugt08* and *BXugt09*, *BXugt11* and *BXugt12*, *BXugt13* and *BXugt14*, *BXugt15* and *BXugt16*, *BXugt17* and *BXugt18*, *BXugt31* and *BXugt32*, as well as *BXugt45* and *BXugt46*. In addition, *BXugt34*, *BXugt35*, *BXugt36*, *BXugt37* and *BXugt38* formed a tandemly arranged gene cluster. Further collinearity analysis indicated that no gene duplication events were detected within the *UGT* gene family of *B. xylophilus*, and the overall frequency of gene duplication in the *B. xylophilus* genome was relatively low compared with that in other species or other gene families (**Supplementary Figure 1**).

### 3.2 Phylogenetic analysis of *UGT* genes in *B. xylophilus* and *C. elegan*

**Figure 2:**
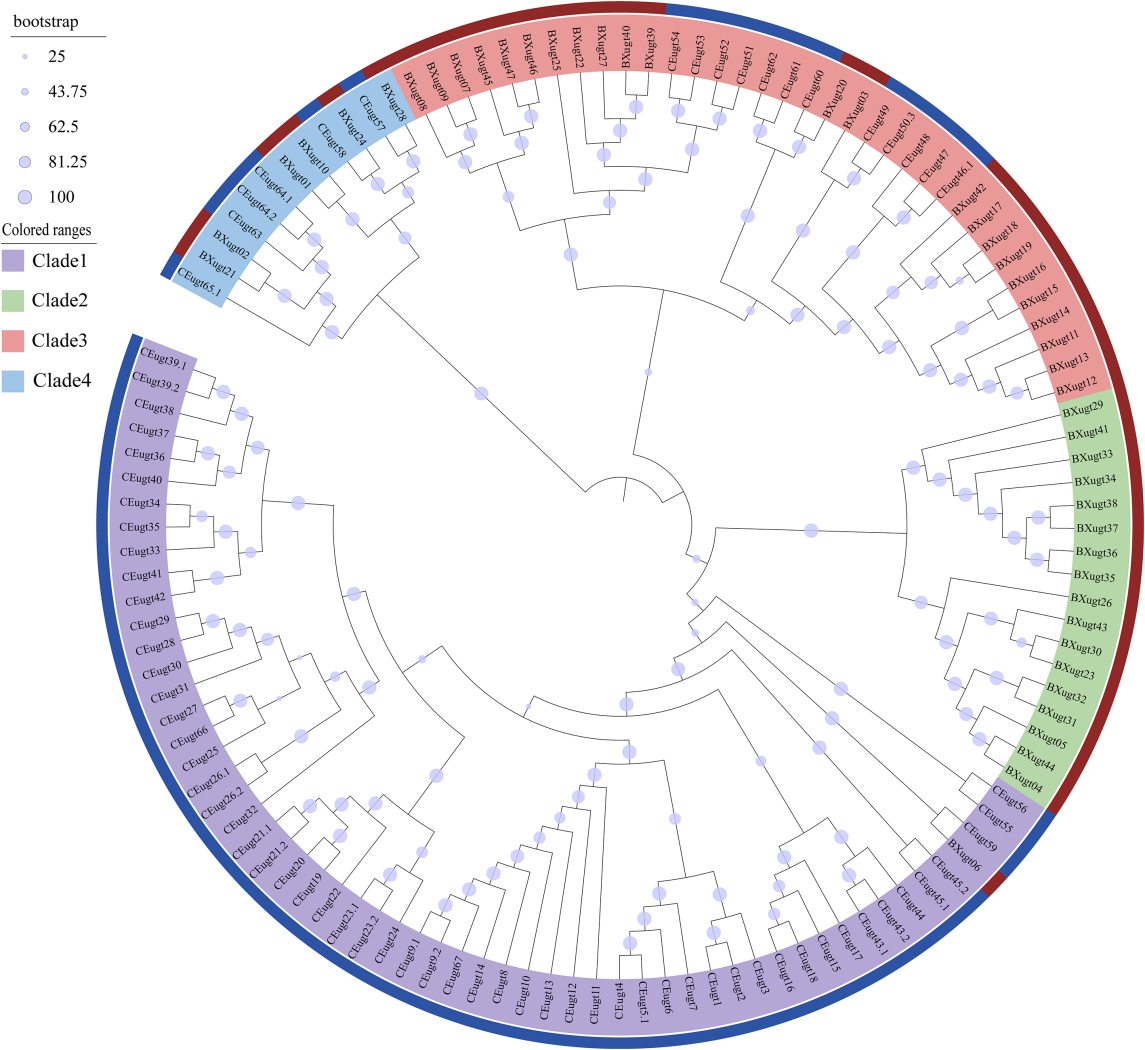
Phylogenetic tree of the *UGT* gene family in the *B. xylophilus* and *C. elegans*. Multiple sequence alignment was performed using MAFFT with default parameters. The maximum likelihood (ML) tree was constructed using IQ-TREE under the best-fit substitution model LG+F+R8, with 2000 ultrafast bootstrap replicates to evaluate branch support. The tree is divided into four well-supported major clades (Clade 1–Clade 4), which are highlighted with distinct background colors. Dark blue bars indicate *UGT* genes from *C. elegans*, whereas reddish-brown bars indicate those from the *B. xylophilus*.

**Figure 3:**
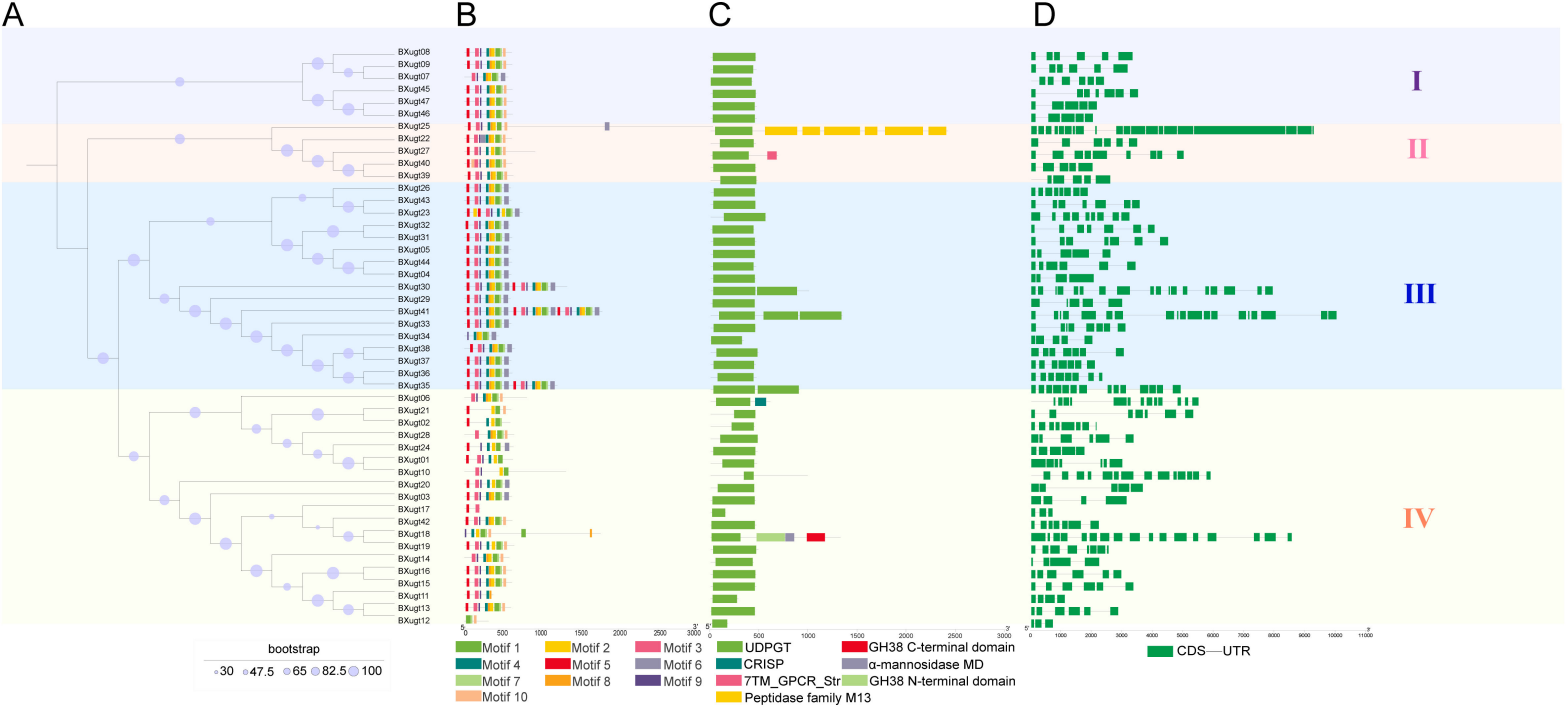
Evolutionary analysis of *UGT* genes in *B. xylophilus*. **(A)** Maximum-likelihood phylogenetic tree of *UGT* genes in *B. xylophilus*, The ML tree was constructed using IQ-TREE under the best-fit substitution model LG+R7, with 2000 ultrafast bootstrap replicates to evaluate branch support. **(B)** Conserved motif analysis, different colored boxes represent distinct motifs (Motif 1–10). **(C)** Domain architecture. Different colored boxes represent specific domains, UDPGT (UDP-glucoronosyl and UDP-glucosyl transferase), GH38 C-terminal domain (Glycosyl hydrolases family 38 C-terminal domain), CRISP (Cysteine-rich secretory protein family), Peptidase family M13, α-mannosidase MD (Alpha mannosidase middle domain), GH38 N-terminal domain (Glycosyl hydrolases family 38 N-terminal domain), 7TM_GPCR_Str (Serpentine type 7TM GPCR chemoreceptor Str). **(D)** Predicted functional domains. Green boxes indicate coding sequences (CDS), and thin lines indicate untranslated regions (UTR). Groups I–IV (labeled on the right) correspond to the four major clades in the phylogenetic tree **(A)**.

Maximum likelihood (ML) phylogenetic tree showed that all *UGT* genes were divided into four major clades (Clades 1–4) (**Figure 2**). Clade 1 comprised 58 genes, mainly from *C. elegans* (57 genes) with only one from *B. xylophilus*. Clade 2 contained 17 genes, all belonging to *B. xylophilus*. Clade 3 consisted of 12 C*. elegans UGT* genes and 23 *B. xylophilus UGT* genes, while Clade 4 included 12 *UGT* genes, with 6 from *C. elegans*. *B. xylophilus UGT* genes were mainly distributed in Clades 2 and 3, whereas *C. elegans UGT* genes were predominantly clustered in Clade 1, indicating distinct phylogenetic distributions of *UGT* genes between the two nematode species (**Figure 2**). We also performed a separate phylogenetic analysis of the 47 *UGT* genes (**Figure 3A**), and the result shows that these genes clustered into four distinct clades (Clades I–IV), indicating a clear evolutionary divergence among the *UGT* family. Genes within the same clade shared relatively high sequence similarity, suggesting potential functional conservation, while those located in different clades exhibited greater sequence divergence, implying functional diversification.

### 3.3 Prediction of domains, motifs of *UGT* genes in *B. xylophilus*

Based on all UGT protein sequences of *B. xylophilus*, ten conserved motifs were identified using the MEME suite, and their distributions were visualized according to the phylogenetic classification (**Figure 3**). The results revealed clear differences in motif composition and arrangement among the four clades (Clades I–IV). UGT proteins in Clade I exhibited relatively conserved motif patterns, with similar motif order and organization across most members. Clade II showed a distinct but comparatively consistent motif structure, while Clades III and IV displayed higher variability, including differences in motif number and positional shifts. These findings indicate that, although *B. xylophilus* UGTs share several conserved structural features, considerable structural divergence has occurred among different phylogenetic clades. To further characterize the functional features of the *UGT* gene family in *B. xylophilus*, conserved domain analysis was performed on all 47 UGT protein sequences (**Figures 3B, C**). The results showed that all 47 genes contained the typical UDP-glucoronosyl and UDP-glucosyl transferase domain (PF00201). This domain is the signature motif of the *UGT* gene family and is responsible for catalyzing glycosylation reactions between substrates and UDP-sugar molecules, highlighting its key role in metabolic regulation and detoxification processes. Interestingly, several *UGT* genes contained additional functional domains besides the core UGT domain, suggesting potential functional diversification among family members.

*BXugt25* contained a Peptidase family M13 domain, indicating possible involvement in protein or peptide processing and degradation (Bianchetti et al., 2002); *BXugt27* contained a Serpentine type 7TM GPCR chemoreceptor domain, implying a potential role in signal perception or transduction (Jacoby et al., 2006); *BXugt06* contained Cysteine-rich secretory protein (CRISP) family domains, which are typically associated with secreted proteins, ligand binding, or defense-related functions (Gibbs et al., 2008). *BXugt18* contained Glycosyl hydrolases family 38 C-terminal and N-terminal domains, as well as an Alpha mannosidase middle domain, suggesting potential participation in carbohydrate metabolism and glycoprotein modification (Cobucci-Ponzano et al., 2010). Prediction of the 3D structures of UGT proteins in *B. xylophilus* via AlphaFold3 revealed that most UGT proteins exhibit typical UGT family folding characteristics (consistent with the conserved domain architecture of glycosyltransferases) (**Supplementary Figure 2**). In contrast, the structures of BXugt10, 18, 25, 30, 35, and 41 differ significantly from those of other members. Further domain analysis indicated that these proteins have undergone domain tandem or domain gain events. This structural differentiation suggests that they may correspond to the evolution of functional specificity. These findings suggest that while the *UGT* gene family in *B. xylophilus* exhibits a highly conserved UDP-sugar transfer function, the presence of additional domains in certain members may confer novel biological functions or specific involvement in signaling and metabolic pathways.

### 3.4 Prediction transcription factors of *UGT* genes in *B. xylophilus*

**Figure 4:**
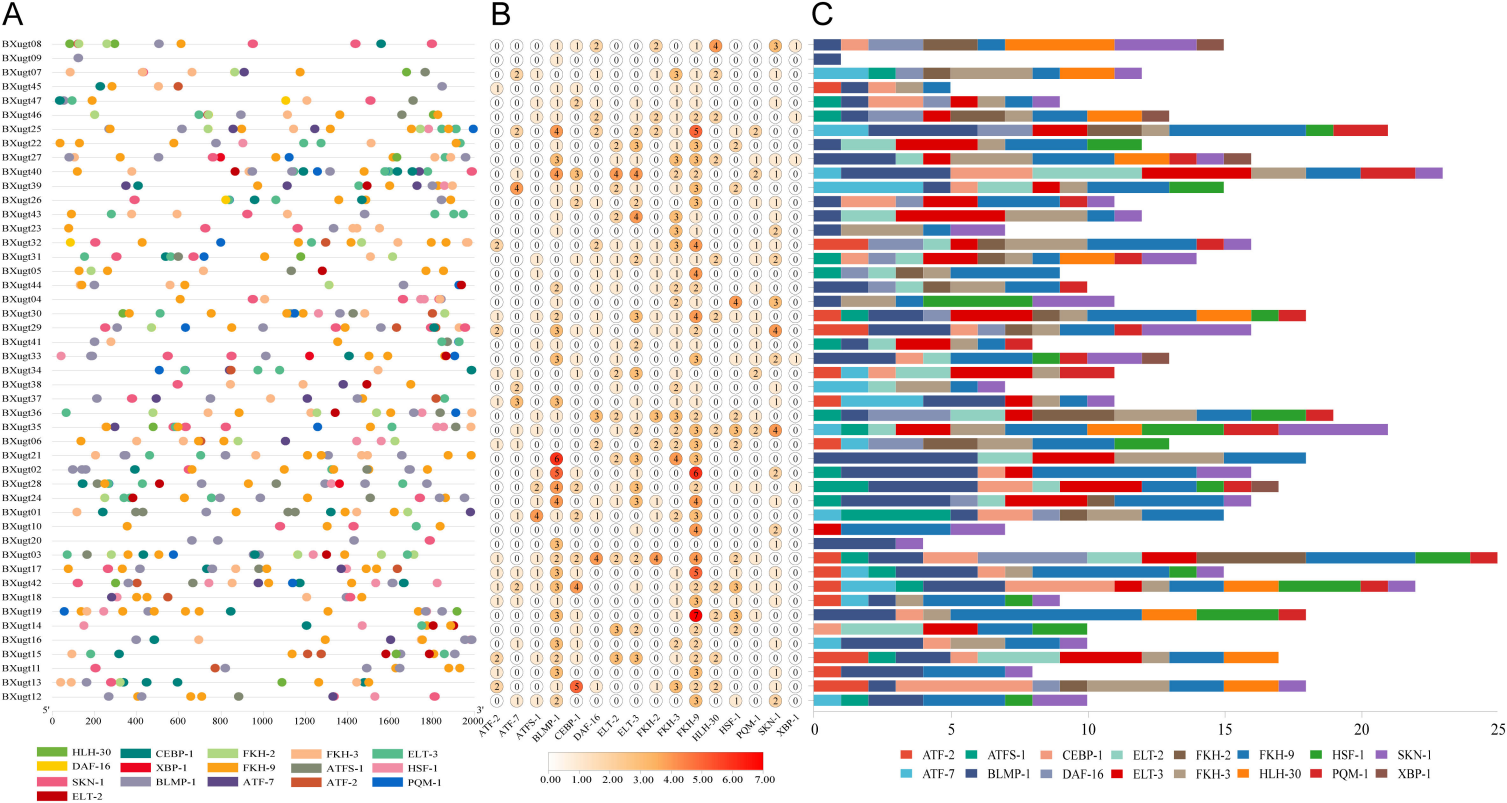
Prediction of transcription factor binding sites within the 2,000 bp upstream regions of *UGT* genes in *B. xylophilus*. **(A)** Genomic distribution of predicted transcription factor binding sites. Each colored dot represents a binding site for a specific transcription factor. **(B)** Heat map of transcription factor abundance. The color gradient represents the relative count of TF binding sites in each UGT gene’s upstream region. **(C)** Stacked bar plot of the total number of transcription factors. Each colored segment corresponds to a distinct TF, illustrating the composition and total count of predicted TF binding sites for each gene. Colored blocks/dots correspond to 16 transcription factors, with consistent color mapping across **(A, C)**.

Analysis of transcription factors associated with *UGT* genes in *B. xylophilus* indicates that many of these regulators are involved in stress responses, detoxification, and metabolic control (**Figure 4**). Notably, SKN-1 is a key regulator of phase II detoxification and oxidative-stress responses (Blackwell et al., 2015), while DAF-16/FOXO integrates metabolic regulation with stress resistance (Lin et al., 2018). In addition, CEBP-1 has been implicated in intestinal immune defense and stress surveillance (McEwan et al., 2016), BLMP-1 functions in developmental and stress-associated transcriptional programs (Huang et al., 2014), and the forkhead transcription factor FKH-9 contributes to endoplasmic reticulum homeostasis during infection (Tillman et al., 2018). These findings support the view that *UGT* gene expression in *B. xylophilus* is under coordinated regulation by stress- and metabolism-related signaling pathways. In addition, several transcription factors, including HSF-1, ELT-2, ELT-3, and the ATF family (ATF-2, ATF-7, and ATFS-1), were predicted, indicating potential links to heat-shock responses, intestinal gene regulation, and the unfolded protein response (UPR). HSF-1 is a central regulator of heat-shock–induced gene expression and cellular proteostasis, while ELT-2 functions as a major GATA transcription factor governing intestinal differentiation and stress-responsive transcriptional programs (Singh and Aballay, 2006; Bouchier-Hayes et al., 2010; Mann et al., 2016). ATF-7 and ATFS-1 are involved in immune and mitochondrial stress signaling, respectively, and mediate transcriptional responses to diverse environmental and cellular stresses (Shivers et al., 2010; Yang et al., 2022). Several transcription factors with lower predicted frequencies, including HLH-30, PQM-1, and XBP-1, were also identified. HLH-30 regulates nutrient-responsive autophagy pathways (Lapierre et al., 2013), PQM-1 contributes to metabolic and stress-induced transcriptional regulation (Tepper et al., 2013), and XBP-1 is a key regulator of the endoplasmic-reticulum–stress–associated UPR (Richardson et al., 2010; Pellegrino et al., 2014). Taken together, these results suggest that *UGT* genes in *B. xylophilus* are subject to multilayered transcriptional control, reflecting a complex regulatory network that likely supports nematode adaptation to fluctuating environmental conditions.

### 3.5 Stress response and infection-stage expression pattern of *UGT* genes in *B. xylophilus*

**Figure 5:**
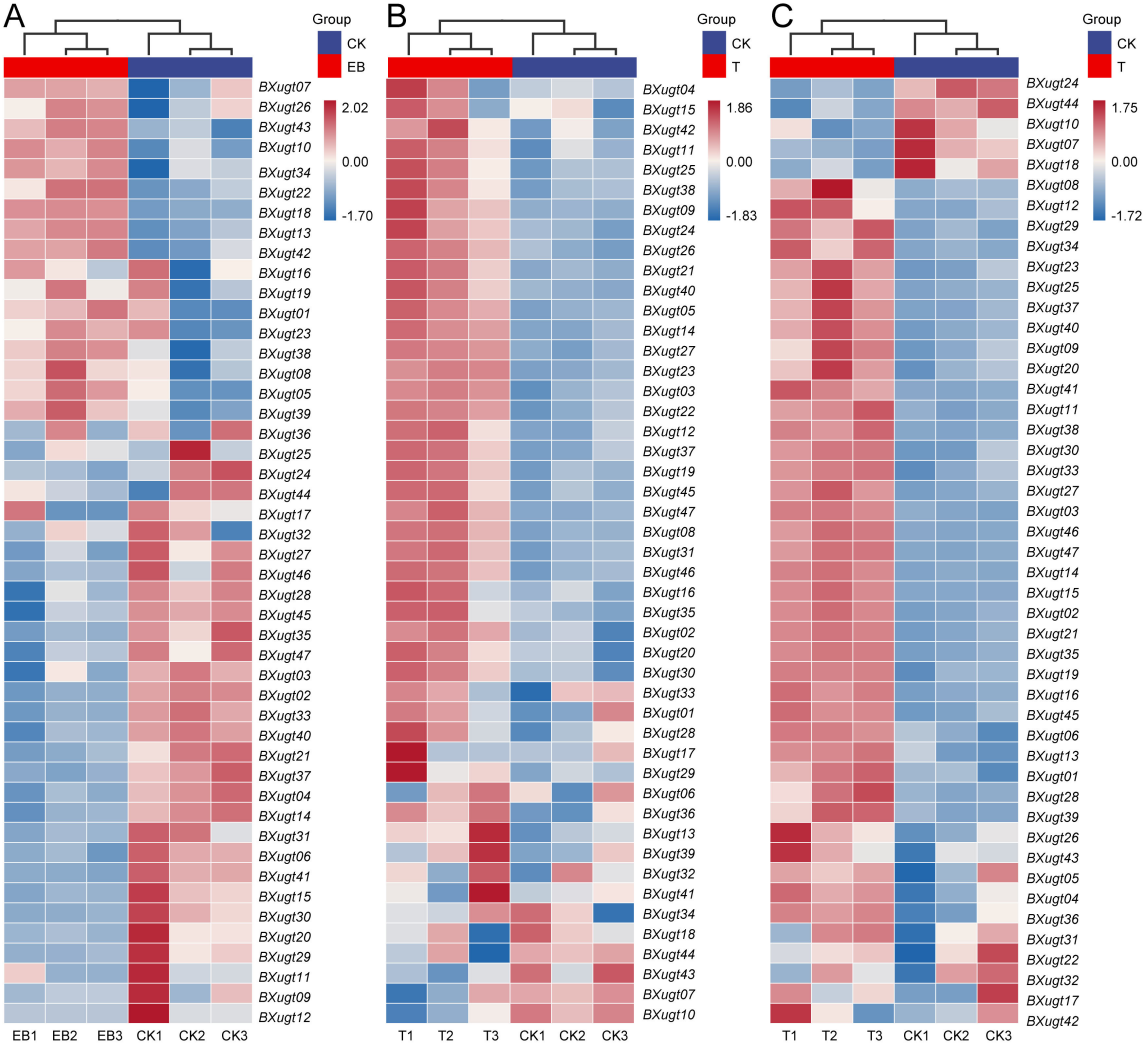
Heatmap of stress response and infection-stage expression levels of *UGT* genes in *B. xylophilus.***(A)** Heatmap of *UGT* genes in *B. xylophilus* under emamectin benzoate stress, EB1–EB3 represent the treatments with emamectin benzoate (group EB), while CK1–CK3 represents acetone (the solvent of emamectin benzoate) control (group CK). **(B)** Heatmap of UGT genes in *B. xylophilus* under tetramycin B3 stress, T1–T3 represent the treatments with emamectin benzoate (group T), while CK1–CK3 represents the sterile water control (group CK). **(C)** Heatmap of *UGT* genes in *B. xylophilus* under host infection period, T1–T3 represent the host infection stage (group T), while CK1–CK3 represents the stage cultured on *Botrytis cinerea* under laboratory conditions (group CK).

To investigate the specific roles of *UGT* genes under pesticide stress and host-derived stress during the infection phase, we analyzed RNA-Seq data from *B. xylophilus* samples subjected to emamectin benzoate treatment, tetramycin treatment, and the infection stage (**Figure 5**). Under the stress of emamectin benzoate, compared with the control group treated with sterile water, 8 *UGT* genes were significantly up-regulated, among which *BXugt18* showed the highest expression level (**Supplementary Figure 3A**). Under the stress of tetramycin B3, 10 *UGT* genes exhibited an up-regulation trend, with *BXugt12* having the highest expression level (**Supplementary Figure 3B**). During the host infection stage, a total of 31 *UGT* genes were up-regulated; among them, the up-regulation of *BXugt46* was the most significant, with its expression level increased by more than 28-fold (**Supplementary Figure 3C**). The expression data of the infection stage indicated that the *UGT* gene family was significantly activated to respond to host-derived environmental stress, and *BXugt46* might act as a key gene in coping with such stress. Notably, 15 out of 17 *UGT* genes in Clade 2 of the phylogenetic tree (a *B. xylophilus*-specific clade) were specifically expressed during the infection stage, suggesting that this clade plays a unique role in the parasitic process of *B. xylophilus*. The enhanced expression of these genes may facilitate the detoxification of host secondary metabolites, thereby supporting the successful colonization and survival of *B. xylophilus* within the host. These results demonstrated that the *UGT* genes of *B. xylophilus* responded extensively to both xenobiotic stress (pesticides) and host-induced stress, highlighting their crucial roles in environmental adaptation, chemical defense, and parasitic fitness.

To verify the accuracy of the transcriptome data, this study employed RT-qPCR technology to detect the genes up-regulated after pesticide treatments and the top 10 genes with the highest up-regulation levels during the infection stage (**Supplementary Figures 4–6**). The results showed that the expression trends of most candidate genes were highly consistent with those of the RNA-Seq profiles, confirming the reliability of the transcriptome dataset. It is worth noting that both *BXugt05* and *BXugt08* were significantly expressed under both pesticide treatments and during the host infection stage, which further highlighted their importance in the detoxification process of *B. xylophilus*.

## 4 Discussion

UGTs represent the largest subgroup of glycosyltransferase families and are widely distributed in fungi, bacteria, plants and animals. They participate in diverse biological processes such as xenobiotic detoxification, endogenous metabolite regulation, and biological interactions by catalyzing glycosylation reactions. Although UGTs play crucial roles in the detoxification processes, a comprehensive identification and systematic analysis of the *UGT* genes in *B. xylophilus* have not yet been accomplished. This study employed multiple technical approaches to characterize these genes and predict their functions. A total of 47 members of the *UGT* gene family were screened out, and these genes exhibited extensive diversity in terms of phylogenetic characteristics, sequence length, chromosomal localization, gene structure, transcription factor binding properties, and specific expression patterns. Based on the phylogenetic analysis of the *UGT* gene family between *B. xylophilus* and *C. elegans*, a total of four unique clades were identified, members within the same clade are likely to share similar structural and functional characteristics. In contrast to the free-living nematode *C. elegans*, the *UGT* gene family of *B. xylophilus* exhibits a higher degree of lineage-specific differentiation, which underscores its critical role in mediating detoxification processes and adapting to complex parasitic life histories. This indicates that the *UGT* gene family has undergone species-specific differentiation during the evolution of *B. xylophilus*, which may represent an important evolutionary strategy for adapting to the pine host environment and provide a molecular basis for successful host infection and colonization. However, this study was limited to comparative analyses with *C. elegans* only, and did not include other plant-parasitic nematodes. The phylogenetic characteristics of the *UGT* gene family in *B. xylophilus* relative to other nematode species remain to be further elucidated. These differences are likely closely associated with host specificity, pathogenicity, and life history traits across different plant-parasitic nematodes, representing an important direction for future comparative genomics research.

Collinearity analysis revealed an extremely low proportion of duplicated genes in the *B. xylophilus* genome, and no duplication events were observed within the *UGT* gene family, which is consistent with the genomic characteristics of *C. elegans* (C. elegans Sequencing Consortium, 1998). In *Populus euphratica*, tandem duplication may serve as the primary driver for the expansion of the *PeUGT* family, which stands in sharp contrast to the pattern observed in *B. xylophilus* (An et al., 2025). Tandem duplication is prone to induce functional redundancy of genes and elevate the risk of gene loss mediated by homologous recombination (Hastings et al., 2009). It is proposed that *B. xylophilus* may maintain genomic stability and potentially promote refined functional differentiation of *UGT* genes by avoiding tandem duplication. During host infection, this nematode needs to achieve precise metabolism of diverse substrates, such as pine terpenoids and defensive compounds (Kopaczyk et al., 2020), rather than improving metabolic efficiency by increasing gene copy numbers. Each *UGT* gene may evolve distinct substrate specificity, which not only avoids the resource consumption caused by redundant genes but also enhances adaptation to the complex host-environment interactions. Meanwhile, the complex parasitic life cycle of *B. xylophilus*, which depends on insect vectors and host pine trees (Kirino et al., 2023), may reduce the selective pressure for the rapid amplification of single genes to counter specific stresses. Regarding the tandemly arranged *UGT* genes, we propose that plant-parasitic nematode genomes generally exhibit the characteristic of genomic streamlining. Tandemly arranged *UGT* genes can reduce the redundancy of regulatory elements and improve genomic utilization efficiency. The limited tandem duplication in *B. xylophilus*, merely the tandem array of multiple distinct *UGT* genes, may have constructed an efficient metabolic defense system while circumventing the mutation risks associated with repetitive sequences.

UGTs can catalyze the conjugation of glycosyl groups to small lipophilic molecules, thereby enhancing the water solubility of these molecules and facilitating their excretion. They play crucial roles in xenobiotic detoxification, metabolic regulation, and environmental adaptation in insects and nematodes (Dimunová et al., 2022; Scott, 2026). Insect UGTs have been widely demonstrated to be involved in the detoxification of various pesticides, thereby contributing to insect resistance. They play crucial roles in xenobiotic detoxification, metabolic regulation, and environmental adaptation in both insects and nematodes. The potential roles of *UGT* genes in insecticide resistance have been illustrated in *Aphis gossypii*, *Bemisia tabaci*, *Spodoptera litura*, and *Spodoptera frugiperda* (Pan et al., 2018; Du et al., 2021; Yang et al., 2023; Su et al., 2023). Among parasites, the induction of *UGT* genes in *Haemonchus contortus* is associated with exposure to anthelmintic drugs (Štěrbová et al., 2025). Additionally, *UGT440A1* of *B. xylophilus* has been reported to be involved in locomotion, reproduction, and pathogenicity (Wang et al., 2022). In *C. elegans*, *UGT* genes exert functions in glycosylation and detoxification (Asif et al., 2024). These findings indicate that *UGT* genes serve as a universal biochemical defense system for invertebrates against various chemical challenges. Analysis of *UGT* gene-associated transcription factors revealed that their expression is coordinately regulated by multiple signaling pathways involved in stress response, detoxification, metabolism, and immunity, forming a complex and multilayered transcriptional regulatory network. These findings provide important insights into the molecular regulatory mechanisms underlying *UGT* genes in environmental stress adaptation and host interaction in *B. xylophilus*. When *B. xylophilus* is exposed to β-pinene released by host pine trees, *UGT* genes are also significantly induced (Li et al., 2020). This indicates that the nematode employs *UGT* genes to initiate detoxification responses against host-derived xenobiotics. Transcriptome analysis in this study revealed that multiple *UGT* genes were up-regulated under pesticide stress and during the infection stage, which is consistent with the *UGT* activation patterns observed in nematodes and insects exposed to chemical stressors. Moreover, the expression patterns of *UGT* genes varied across different stress stages, demonstrating that *UGT* genes may have evolved distinct substrate specificities. From a practical perspective, the involvement of *UGT* genes in *B. xylophilus*’ response to both host defense substances and pesticides may affect the efficacy of chemical control strategies against pine wilt disease. The UGT-mediated detoxification mechanism may enhance the nematode’s tolerance to xenobiotics; subsequent elucidation of its specific functional mechanisms will not only help understand the biological characteristics of *B. xylophilus* but also provide important targets for the development of novel nematicides. While this study was primarily based on descriptive bioinformatic analyses without direct molecular functional validation of *UGT* genes, which to some extent constrains the robustness of our biological interpretations, it nevertheless represents the first comprehensive characterization of the *UGT* gene family and their potential biological functions in *B. xylophilus*. Our findings provide novel molecular insights into the mechanisms underlying host infection and stress responses in this nematode.

## Data Availability

The datasets presented in this study can be found in online repositories. The names of the repository/repositories and accession number(s) can be found in the article/supplementary material. The raw sequencing data generated from gene analyses in this study have been deposited in the NCBI database (PRJNA1401932).
