# Supplementary material for: Genome-wide characterization of the Bursaphelenchus xylophilus UGT gene family and its potential roles in detoxification and host interaction
Source: Front Plant Sci. 2026 Apr 10;17:1786843. doi: 10.3389/fpls.2026.1786843 (PMC13106581; doi:10.3389/fpls.2026.1786843)
Supplement: Supplementary file 1 [file DataSheet1.docx]

Supplementary Material

# Supplementary Figures and Tables

## Supplementary Figures


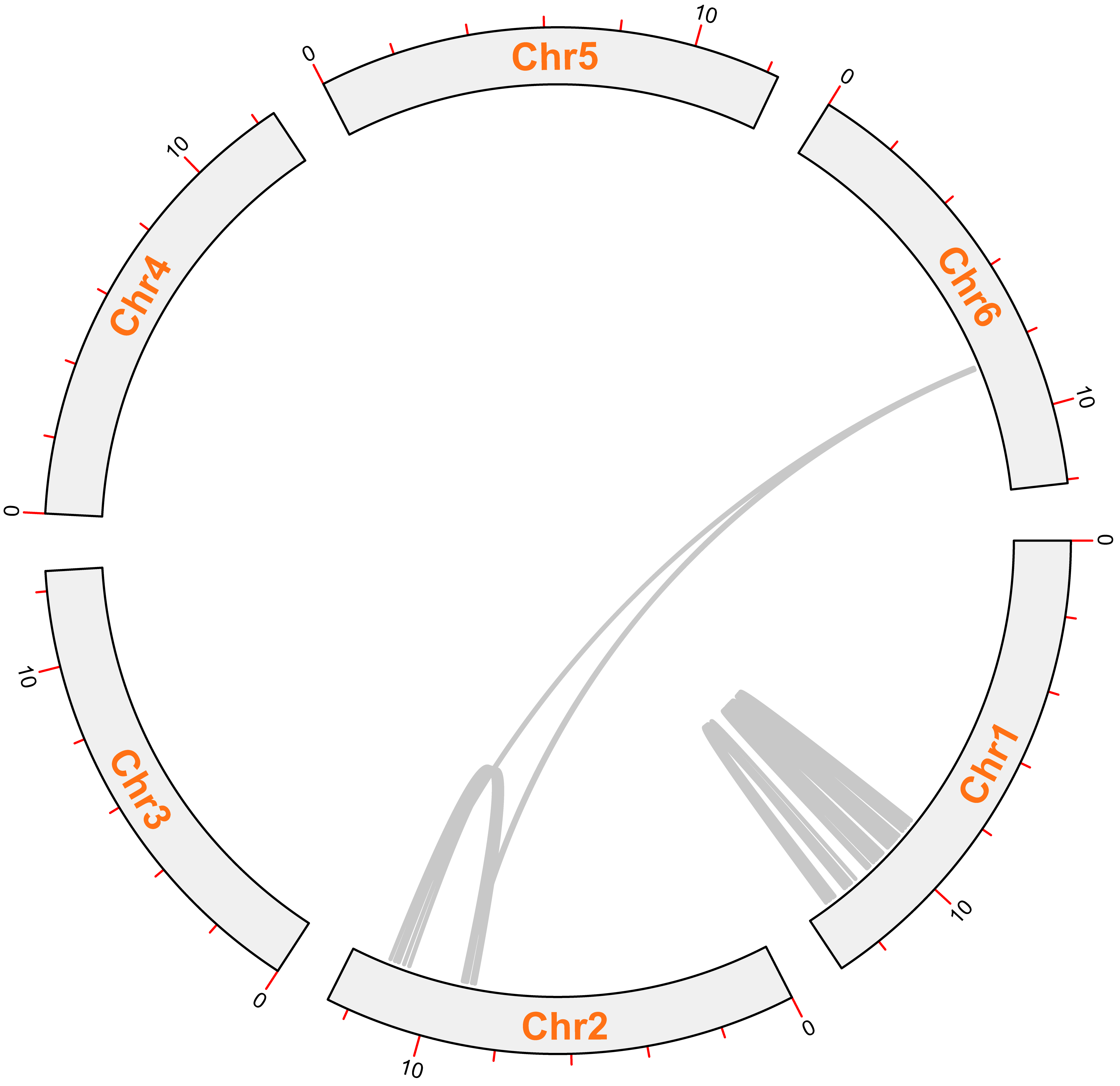


**Supplemental Figure 1.** Intraspecific collinearity analysis of genes in the B. xylophilus. The outer circle represents the six chromosomes of B. xylophilus, and the gray lines inside the circle indicate collinear gene pairs. No collinear *UGT* gene pairs were detected and therefore are not highlighted in the figure.


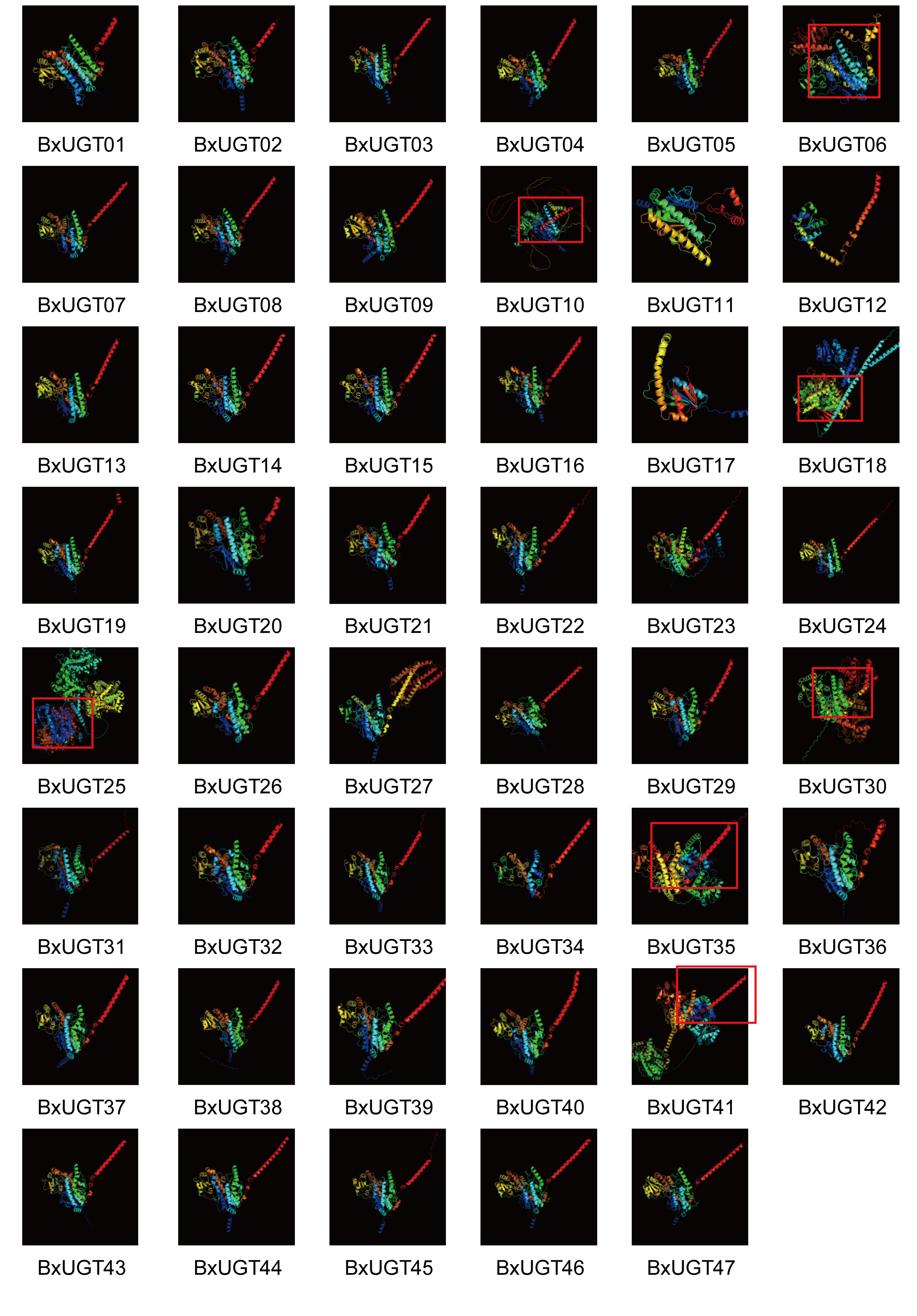


**Supplemental Figure 2.** Predicted 3D structures of 47 UGT proteins from *B. xylophilus* using AlphaFold3. The UGT domains predicted in this study are highlighted in red and enclosed within red boxes.


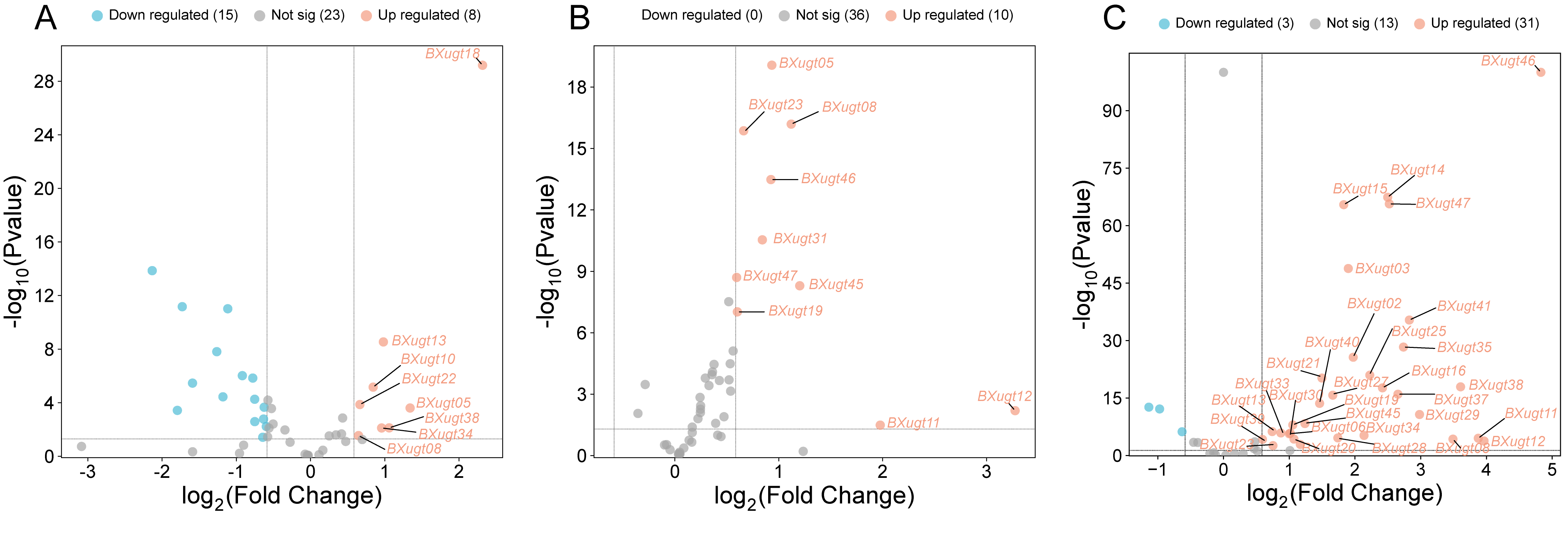


**Supplemental Figure 3.** Volcano plot of *UGT* gene expression identified by transcriptomic analysis. (A) Volcano plot of *UGT* gene expression under emamectin benzoate stress, showing fold change (log_2_ (Fold Change)) and statistical significance (−log_10_(Pvalue)). (B) Volcano plot of *UGT* gene expression under tetramycin B3 stress. (C) Volcano plot of *UGT* gene during the infection stage.


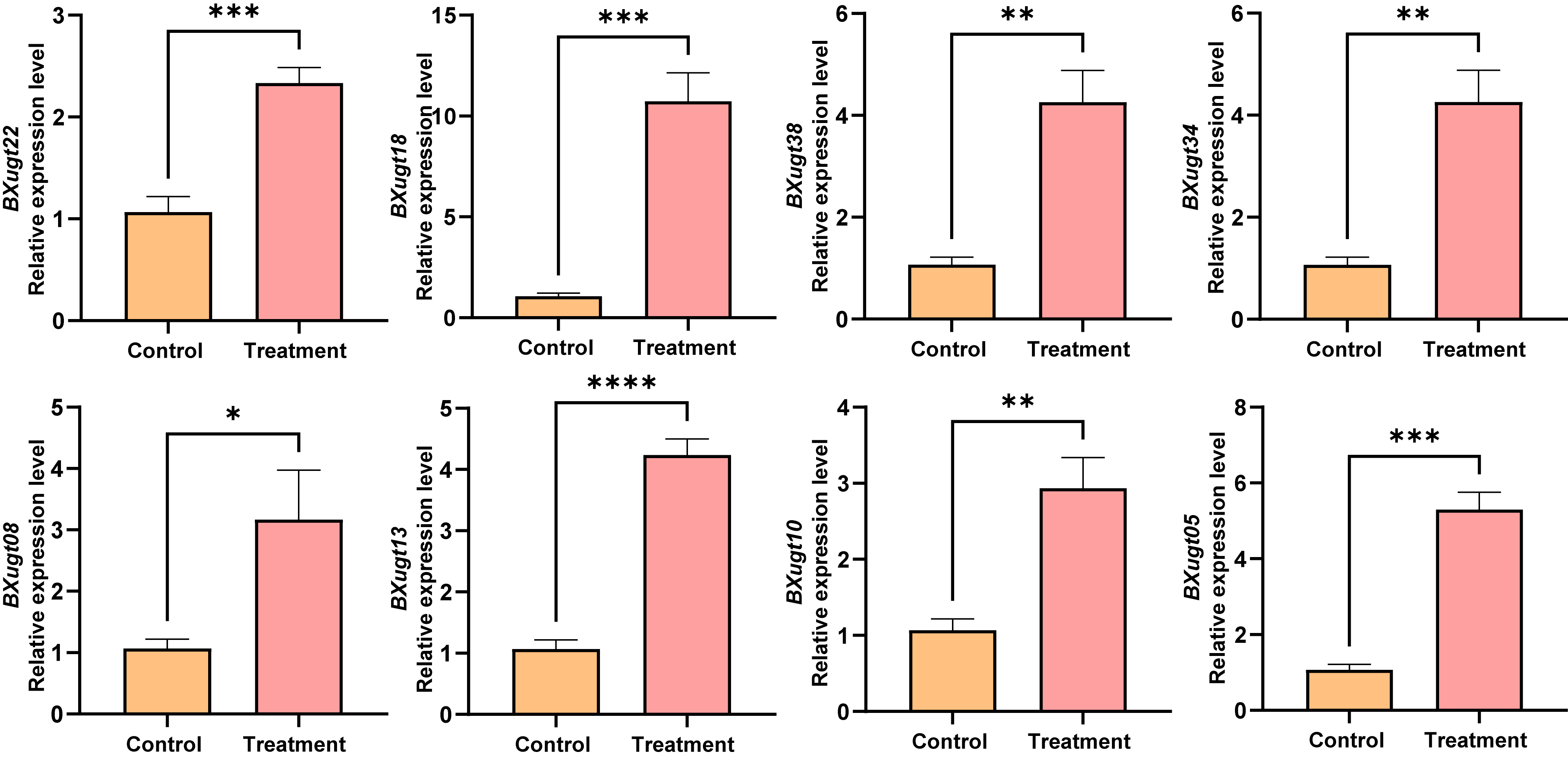


**Supplemental Figure 4.** RT-qPCR validation of the upregulated *UGT* genes under emamectin benzoate stress with * indicating P < 0.1, ** indicating P < 0.01, *** indicating P < 0.001, ****indicating P < 0.0001


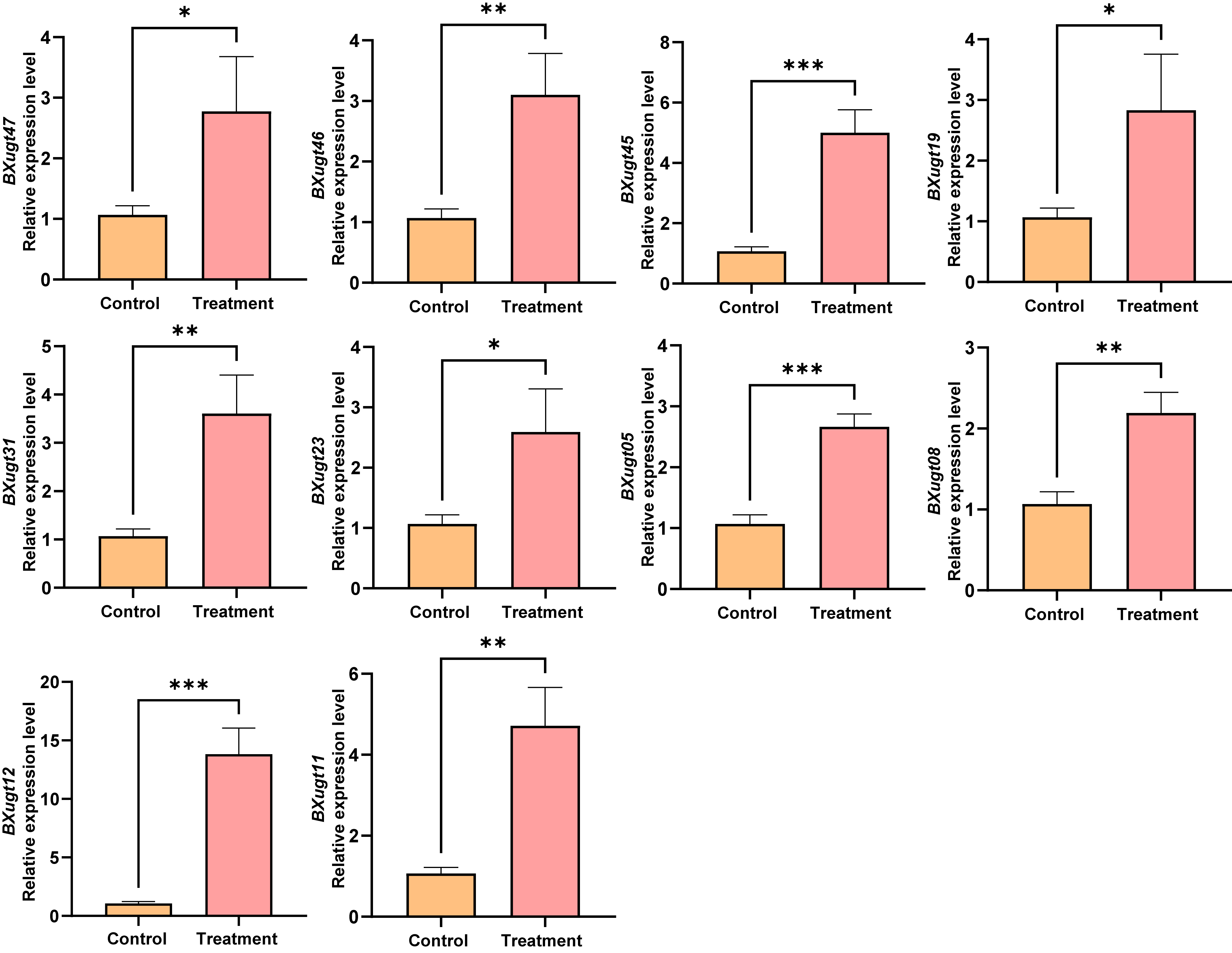


**Supplemental Figure 5.** RT-qPCR validation of the upregulated *UGT* genes under tetramycin B3 stress with * indicating P < 0.1, ** indicating P < 0.01, *** indicating P < 0.001.


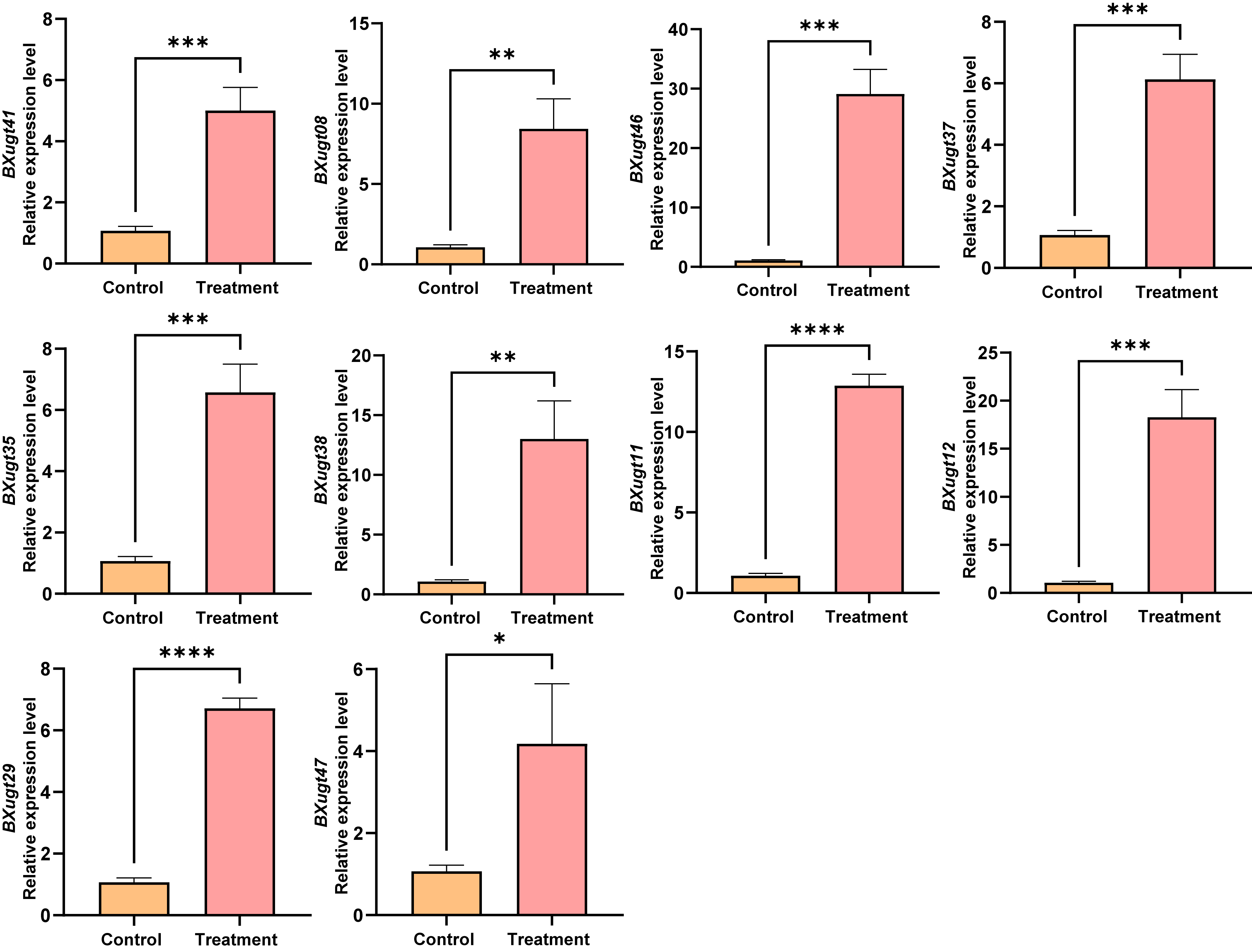


**Supplemental Figure 6.** RT-qPCR validation of the top 10 upregulated *UGT* genes during the infection stage, with * indicating P < 0.1, ** indicating P < 0.01, *** indicating P < 0.001, **** indicating P < 0.0001.

## Supplementary Tables

**Supplemental Table 1.** Primers required for RT-qPCR

| **Primer name** | **Sequence (5'-3')** |
| --- | --- |
| BXugt05-F | TAGTTTGGTGCCAGGACGTC |
| BXugt05-R | TCGCCATCGCTCTTCTCATC |
| BXugt08-F | TCCAGGACCGAGGACATCAT |
| BXugt08-R | TCGCTGAAGACGTCGTTGAA |
| BXugt10-F | TACCCCGAATCCCTACCGTT |
| BXugt10-R | CTCCGAGTTATCCGTGTCCG |
| BXugt11-F | TTGGCGGATTTGGGATGGAT |
| BXugt11-R | TCCTCCTGTATGTCTGCGGA |
| BXugt12-F | GGACGGAGGAATTCGAAGGG |
| BXugt12-R | AGGCGTTGTTCTCCTGTCTG |
| BXugt13-F | TTCGATTGGCCAGAACGACA |
| BXugt13-R | GCGGATGCTTGCATTCGATT |
| BXugt18-F | ATCCTGGTGGAGACTCCCTC |
| BXugt18-R | ATTTGAGCTTCCTGAGGGCC |
| BXugt19-F | TGTGAAACACGCCAAGCATG |
| BXugt19-R | ACACGCCTCCACTTTTCGAT |
| BXugt22-F | CCCGCAGAAACAGTTGTTGG |
| BXugt22-R | TCAAATGCAACGCAATGCCA |
| BXugt23-F | CCGCTGAAGCTGCCAAATTT |
| BXugt23-R | TCGGCCAACTTGTCGATGAA |
| BXugt29-F | ATTGGCGGAATTACCCAGCA |
| BXugt29-R | AGTCTCGGCCAAGATCTCCT |
| BXugt31-F | TTTCCCCGGAAGAACGAGTG |
| BXugt31-R | GCTGAGTTTACGACCGTCCA |
| BXugt34-F | GTGGCTATGCAATTGCGGAG |
| BXugt34-R | TAAGCAACGCCTTGACTCGT |
| BXugt35-F | CACCACAAGCCGAGCTTCTA |
| BXugt35-R | GTTTTCCGTTTTGAGCCGCT |
| BXugt37-F | GATTGTTCGACCATGCGAGC |
| BXugt37-R | CAAGCCTCTCCCATCGTTGT |
| BXugt38-F | GGACTCAACAGTGTCAGCGA |
| BXugt38-R | CCAGTCCTTTTTCTTGCGCC |
| BXugt41-F | AGTGCCAACAATTTGCGTCC |
| BXugt41-R | AGCAATTTTCCCAACGCGTC |
| BXugt45-F | CCGCTGAAGCTGCCAAATTT |
| BXugt45-R | TCGGCCAACTTGTCGATGAA |
| BXugt46-F | CATTTGGGTCTGTTGCGCAA |
| BXugt46-R | GGAGCCATCTCTCGACATGG |
| BXugt47-F | CATTTGGGTCTGTTGCGCAA |
| BXugt47-R | GGAGCCATCTCTCGACATGG |
